# RAM-MSA: an anytime memory-bounded method for exact multiple sequence alignment using path finding

**DOI:** 10.1093/bioadv/vbag170

**Published:** 2026-06-16

**Authors:** Jue Wang, Fumihiko Ino

**Affiliations:** Graduate School of Information Science and Technology, The University of Osaka, Suita, Osaka 565-0871, Japan; Graduate School of Information Science and Technology, The University of Osaka, Suita, Osaka 565-0871, Japan

## Abstract

**Motivation:**

Multiple sequence alignment (MSA) is a crucial process in bioinformatics, essential for understanding functional or structural relationships among DNA or protein sequences. As the number of sequences increases, existing exact MSA methods, which produce algorithmically optimal alignments, suffer from exponentially increasing memory consumption. Existing heuristic MSA methods, on the other hand, rapidly compute alignments on large sequence sets by sacrificing their accuracy. Current approaches remain insufficient, underscoring the necessity of a novel approach that bridges the gap between heuristic and exact MSA methods. Specifically, there is an urgent need for an exact MSA approach that enables users to obtain the best possible alignment within their available computation time.

**Results:**

We propose Recursive Anytime Memory-bounded MSA (RAM-MSA), a novel exact MSA method that manages exponential memory demands within a limited memory space. The proposed method promptly generates an initial MSA result and continuously outputs alignments with higher accuracy, ultimately providing an exact alignment. Experimental evaluations demonstrate that RAM-MSA reduced memory usage to 62.51% compared to a state-of-the-art exact MSA method. In terms of anytime performance, the proposed method generates an initial alignment with an objective score of 0.96 in a comparable time to the heuristic methods, and continues to improve the alignment until the exact result is obtained. Unlike existing path-finding-based methods, which support only linear gap penalties, RAM-MSA also handles affine gap penalties. This study provides a systematic quantification of the discrepancy between algorithmically optimal alignments and structurally-derived reference alignments.

**Availability and implementation:**

Source code is available at https://github.com/luxwj/RAM-MSA.

## 1 Introduction

Multiple sequence alignment (MSA) plays a crucial role in various sequence analysis applications. Similar symbols in input sequences are aligned to reveal the evolutionary or structural relationships among the sequences. In protein structure prediction, MSA results are necessary to improve the accuracy of the output structure (Jumper *et al.* 2021). For epidemic research, tracing viral evolution heavily relies on MSA methods (Katoh and Standley 2021).

MSA algorithms represent biological sequences as strings and generate alignments by inserting gaps. These alignments are scored using a substitution matrix that assigns a score to each pair of symbols. An exact MSA method, such as a generalized Needleman-Wunsch algorithm extended to high-dimensional spaces (Needleman and Wunsch 1970), produces an algorithmically optimal alignment. Such an optimal alignment achieves the highest possible score under the given substitution matrix. However, algorithmic optimality may diverge from biological correctness. To evaluate the biological significance of the generated alignments, the results should be compared against the reference alignments in the benchmarks. Notably, all MSA approaches mentioned in this paper are global MSA methods, which align the sequences across their entire length.

Most of the existing MSA approaches are heuristic methods and generate approximate alignments because MSA problems are known to be NP-complete (Wang and Jiang 1994). However, existing heuristic methods often fail to produce sufficiently accurate alignments on real-world sequence sets (Chowdhury and Garai 2017).

In contrast, exact MSA approaches are preferred in accuracy-critical applications. Current exact MSA methods are unable to handle large-scale sequence sets due to the exponentially growing memory consumption (Bani Baker *et al.* 2024, Hosseininasab and Van Hoeve 2021, Lin *et al.* 2007, Sundfeld *et al.* 2018). Dynamic programming (DP) based methods and path-finding methods require $O(l^{n})$ and $O(2^{nl})$ memory, respectively, where *n* is the number of sequences and *l* is the maximal length of sequences. Once the available memory is exhausted, these methods simply terminate without alignment results. Furthermore, the scoring scheme of existing path-finding methods is limited to linear gap penalties, whereas popular heuristic methods support the more biology-realistic affine gap penalties.

For large-scale problems where exact solutions may be unattainable, an anytime method allows users to obtain the best possible solution within their available time. Several anytime A* methods have been proposed, including MAWA* (Vadlamudi *et al.* 2010), ARA* (Likhachev *et al.* 2003), and APS (Vadlamudi *et al.* 2016). A problem setting closely related to MSA is the longest common subsequence (LCS), where anytime column search (ACS) has been used to develop anytime approaches (Djukanovic *et al.* 2020). However, extending such approaches to MSA is nontrivial because MSA is a non-uniform cost search problem with complex substitution matrices and gap penalties, whereas LCS is a uniform cost search problem.

To address the limitations of existing heuristic and exact MSA approaches and bridge the gap between them, we propose a novel exact MSA method called Recursive Anytime Memory-bounded MSA (RAM-MSA). Our method is designed based on the A* algorithm (Hart *et al.* 1968), which builds a search tree to convert MSA problems into path-finding problems. To promptly generate an initial MSA result, our method adopts a greedy path-finding strategy. Furthermore, it continuously updates the MSA result by integrating the ACS paradigm into A* search, which eventually outputs an exact alignment. To cope with exponential memory growth, RAM-MSA employs a memory-bound strategy that removes some unexpanded tree nodes when the pre-defined memory threshold is reached during the search process. The pruned nodes are later restored when sufficient memory space becomes available, ensuring the exact solutions.

Our contributions are as follows:

- We propose a recursive MSA approach that refines score estimation in A* search. The proposed approach leverages (n−1)-D MSA results to guide *n*-D tasks, i.e. tasks with *n* input sequences, producing more accurate score estimates than the conventional Carrillo and Lipman (CL) lower bound (Carrillo and Lipman 1988) in most cases. This enhancement adds no extra computational cost beyond the existing exponentially scaling workload $O(2^{nl})$.
- Our approach incorporates the ACS paradigm to realize an anytime property, enabling continuous generation of intermediate MSA results with increasing accuracy, and eventually outputs the exact MSA. We extended the ACS method to accommodate the feature of non-uniform cost search in MSA tasks. The initial alignment reaches up to 98% accuracy with a runtime comparable to heuristic MSA methods like MAFFT (Katoh and Standley 2013).
- A memory-bound strategy is developed to compute large-scale exact MSA problems under limited memory capacity. By temporarily removing selected unexpanded nodes from the search list, the method reduces memory consumption to near-linear levels, whereas conventional path-finding approaches incur exponential growth in list size.
- To the best of our knowledge, our method is the first path-finding-based MSA approach supporting affine gap penalties. Compared to the linear gap penalty model, the affine gap penalty model is more essential for producing biologically realistic alignments. Specifically, protein sequence alignments prefer a long gap to multiple distributed gaps. However, existing path-finding-based exact MSA methods are unable to handle affine gap penalties because doing so would substantially increase workload and memory consumption compared to linear gap penalties.
- We comprehensively evaluated RAM-MSA from both algorithmic and biological perspectives. Specifically, we compared the reference alignments against the exact alignments generated based on the optimal score under a given substitution matrix. This study experimentally demonstrates the discrepancy between algorithmically optimal alignments and structurally-derived reference alignments.

## 2 Background

### 2.1 Multiple sequence alignment algorithms

**Figure 1:**
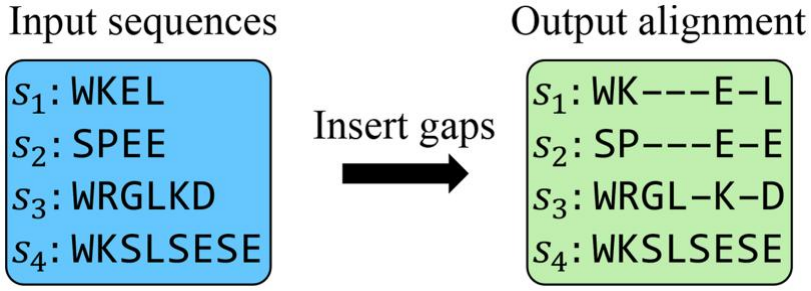
Example of MSA (n=4). The symbol “-” represents a gap.

To process biological sequences with MSA methods, the sequences are often transformed into strings. For protein sequences, each symbol in a string corresponds to one of the twenty standard amino acids. Figure 1 shows an example of MSA, which takes n≥3 sequences as inputs and generates the output alignment by inserting gaps into each sequence. The input sequences may have different lengths, but the output alignment consists of *n* sequences with the same length *l*.

The sum-of-pairs (SP) score serves as the objective function in exact MSA methods. The final output of an exact MSA method is the alignment with the highest SP score. Assuming an alignment *A* contains *n* sequences of equal length *l*, the SP score of *A* is calculated as follows:


(1) $$\text{SP}(A)=\sum_{1\leq i<j\leq n}\sum_{k=1}^{l}s(A[i][k],A[j][k]),$$

Where *A[i][k]* denotes the *k*-th symbol in the *i*-th sequence of the alignment, and s(x,y) is a function calculating the score of two symbols, *x* and *y*, based on a substitution matrix and gap penalties. Common substitution matrices used in protein sequence alignment tasks include BLOSUM (Henikoff and Henikoff 1992), PAM (Dayhoff 1972), and Gonnet (Gonnet *et al.* 1992), which define scores for all 210 possible substitution pairs of the twenty standard amino acids (and their corresponding symbols). Each element in *A* is either a symbol from the alphabet, e.g. one of the twenty amino acids in protein sequences, or a gap, denoted by “-”.

An alignment must satisfy the following constraints: (1) For any two symbols, the *k*-th and *m*-th with k<m, in the same sequence, their corresponding column indices in *A* must preserve the same order. (2) Columns consisting entirely of gaps are not allowed. Let ϕ(A[i],k) denote the column index of the *k*-th (non-gap) symbol in sequence *A[i]*, i.e. the *i*-th sequence in alignment *A*. Let δ(A[i],k)=1 if sequence *A[i]* contains a non-gap symbol in the *k*-th column, and 0 otherwise. An exact MSA method maximizes the SP score subject to the following two constraints:


(2) $$\begin{array}{cc} \max. & \text{SP}(A),\\ s.t. & \forall i\in [1,n],\;\forall k,m\in [1,l]:\phi (s_{i},k)<\phi (s_{i},m)\\ & \Rightarrow \phi (A[i],k)<\phi (A[i],m),\\ & \forall k\in [1,l]:\sum_{i=1}^{n}\delta (A[i],k)\geq 1. \end{array}$$

A gap penalty is applied when either symbol is a gap in s(x,y). The affine gap penalty is a widely used scoring scheme in heuristic MSA methods. It introduces a gap opening penalty *o* and a gap extension penalty *e*. Gap opening and extension refer to the penalties for opening a gap of any length and extending the length of any existing gap by one, respectively. The affine gap penalty is calculated as follows:


(3) $$p_{\text{aff}}=o+e\times m,$$

Where *m* is the length of the gap. Thus, the penalty of a gap-symbol pair, i.e. a pair consisting of a gap and a non-gap, cannot be calculated independently. Instead, the scoring function refers to neighboring pairs to determine the penalty value. Note that no gap penalty is applied to a pair of symbols when both are gaps, and the gap length does not increase.

To simplify score calculation and reduce workload, the affine gap penalty can be replaced with the linear gap penalty by setting the open penalty *o* to 0. In this case, the gap penalty becomes proportional to the gap length, and the penalty is calculated independently for each gap-symbol pair. However, methods using the linear gap penalty tend to produce less accurate alignments because a genetic event often inserts or deletes consecutive residues rather than multiple separated ones (Altschul 1998, Wang *et al.* 2011).

### 2.2 A* Algorithm for MSA

**Figure 2:**
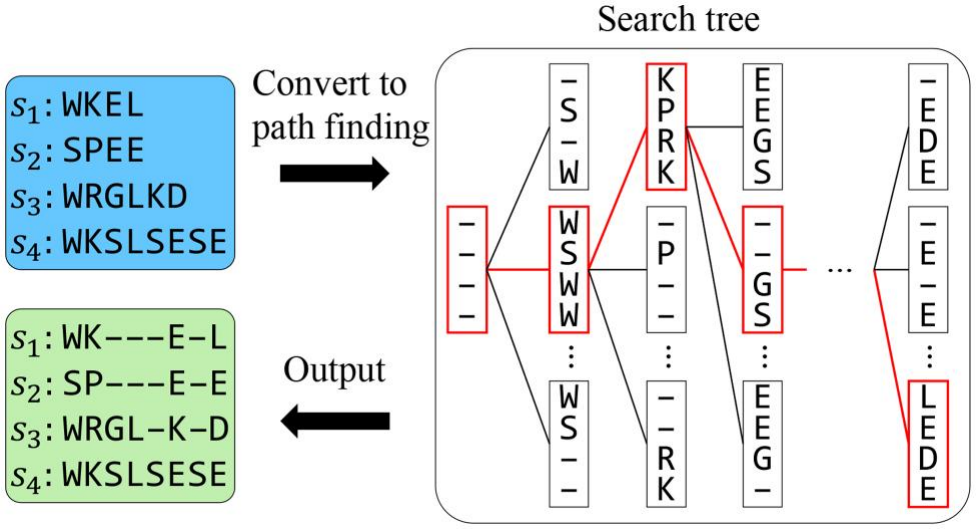
Search tree in the path-finding algorithm for MSA. A node represents a state showing how many symbols from each sequence have been aligned. Red nodes indicate the path corresponding to the output alignment.

Path-finding algorithms, especially the A* algorithm (Hart *et al.* 1968), are widely used for exact MSA computation. Figure 2 shows the search tree structure of such a path-finding algorithm. A node in the algorithm represents a state indicating how many symbols from each sequence have been aligned. For instance, in a task with *n* sequences $\left\{s_{1},s_{2},\ldots ,s_{n}\right\}$, a node includes an *n*-dimensional coordinate array ($c_{1},c_{2},\ldots ,c_{n}$), where each coordinate $c_{i}\;(1\leq i\leq n)$ indicates the current position within the *i*-th sequence. The search begins at a source node, in which all coordinates are zero. The target node is reached when the *i*-th coordinate equals the length of the *i*-th sequence.

The objective function defined in Equations (1) and (2) is reflected as the path score in the A* algorithm, which finds the path with the optimal score (i.e. the shortest path) from the source to the target. Each column in the alignment corresponds to one level of the search tree, and the height of the tree equals the alignment length *l*. Once the search terminates, the alignment is generated by iteratively tracing back from the target through its parent nodes to the source.

Algorithm 1 A* algorithm for MSA

**Input:** A set of sequences $S=\{s_{1},s_{2},\ldots ,s_{n}\}$.

**Output:** The exact alignment *A*[*n*][*l*].

1: Create the source node $p_{s}$ with all-zero coordinates $0_{n}$

2: Open list $L_{\text{open}}=\{p_{s}\}$ // priority queue

3: Closed list $L_{\text{closed}}=\emptyset$ // unordered map

4: **while**  $L_{\text{open}}\neq \emptyset$  **do** 

5:  Current node $p=L_{\text{open}}.\text{pop}()$

6:  **if**  p.is_target()  **then** 

7:   **return** *A* = traceback(*p*) // search succeeded

8:  **end if** 

9:  **if**  $p.\text{gscore}<L_{\text{closed}}.\text{find}(p).\text{gscore}$  **then** 

10:   $L_{\text{closed}}.\text{insert}(p)$

11:   **for** each *q* **in** neighbors of *p* **do** 

12:    **if**  $q.\text{gscore}<L_{\text{open}}.\text{find}(q).\text{gscore}$  **and**  $q.\text{gscore}<L_{\text{closed}}.\text{find}(q).\text{gscore}$  **then** 

13:     $L_{\text{open}}.\text{insert}(q)$

14:    **end if** 

15:   **end for** 

16:  **end if** 

17: **end while** 

18: **return**  A=∅ // search failed

The A* algorithm for MSA is outlined in Algorithm 1. As one of the most efficient path-finding algorithms, A* uses the best-first search strategy that always expands the most promising node. Each node *p* in the search tree maintains a g-score, representing the shortest path score from the source node to *p*. A* computes an h-score for each node, which is a heuristic estimate of the shortest path score from *p* to the target. The f-score, defined as the sum of the g-score and h-score, corresponds to the estimated score of the shortest path passing through *p* from source to target.

The open list contains nodes to expand, sorting by their f-scores in ascending order. A* also uses a closed list to record the expanded nodes, which prevents duplicate node expansion. The open and closed lists are actually a priority queue and an unordered map, respectively, but we continue to refer to them as “lists” for consistency with prior literature.

Each node in the open list stores its (1) coordinate array, (2) g-score, (3) f-score, and (4) traceback information. When using linear gap penalties, the traceback information refers to a bit array that encodes the direction to the parent node. Each node in the closed list is stored as a key-value pair, where the coordinate array serves as the key, and the value is a structure containing (1) the traceback information and (2) the g-score. This data structure corresponds to the existing A* algorithms for MSA methods using linear gap penalties. The data structure for affine gap penalties is introduced in Section 3.4.

The space and time complexities of the A* algorithm are both $O(2^{nl})$ under linear gap penalties. The workload exponentially increases with the number of sequences and the alignment length.

### 2.3 CL Lower bound

**Figure 3:**
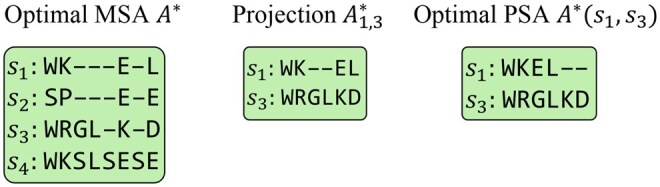
The PSA of a sequence pair, like $s_{1}$ and $s_{3}$, from an MSA is guaranteed to be at least as good as the projection onto that pair in terms of alignment score.

A* search relies on a heuristic function to estimate the path score from an arbitrary node to the target. The CL lower bound provides a simple and efficient approach for the heuristic function (Carrillo and Lipman 1988). As shown in Fig. 3, the projection $A_{1,3}^{*}$ is the two rows of $s_{1}$ and $s_{3}$ extracted from the optimal MSA. The columns with two gaps are removed because they have no contribution to the SP score. The optimal pairwise sequence alignment (PSA) of $s_{1}$ and $s_{3}$ is shown on the right side of Fig. 3. There are two matches (W and L) in the optimal PSA, while the projection has only one match (W). The PSA thus has a lower score than the projection. The CL lower bound is computed on the basis of Equation (4):


(4) $$c(A_{i,j}^{*})\geq c(A^{*}(s_{i},s_{j})),$$

Where 1≤i<j≤n and c(A) is the score of an alignment *A*.

During the A* search of MSA, the CL lower bound of a node can be directly calculated from the cells of the precomputed PSA DP matrices. For instance, three DP matrices $M_{12}$, $M_{13}$, and $M_{23}$ are computed for a 3D MSA task with sequences $s_{1}$, $s_{2}$, and $s_{3}$. Note that the sequences are reversed before the PSAs are computed to allow the method to directly access the DP score from an intermediate node to the target node. Suppose we compute the CL lower bound of a node *p* with coordinates $\left\{x_{1},x_{2},x_{3}\right\}$, then the CL lower bound (or the h-score) of the node is computed as follows:


(5) $$\begin{array}{c} h_{p}=\sum_{1\leq i<j\leq n}c(A^{*}({\hat{s}}_{i},{\hat{s}}_{j}))\\ =\sum_{1\leq i<j\leq n}M_{ij}[l_{i}-x_{i}][l_{j}-x_{j}], \end{array}$$

Where $l_{i}$ is the length of the *i*-th sequence, and ${\hat{s}}_{i}=s_{i}[x_{i}:l_{i}]$ denotes the suffix of $s_{i}$ associated with node *p*. The suffix is a subsequence of $s_{i}$ starting at position $x_{i}$.

## 3 Methods

### 3.1 Recursive MSA approach

**Figure 4:**
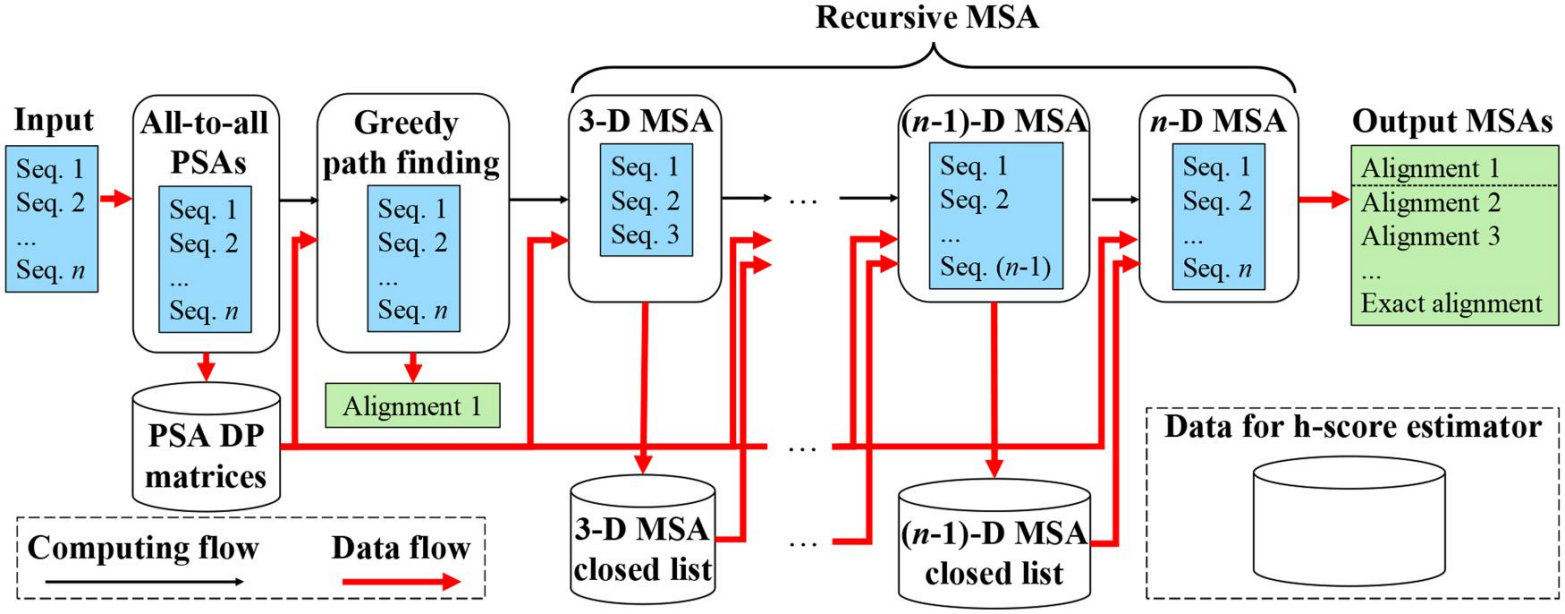
Workflow of the proposed RAM-MSA.

The workload of exact MSA increases exponentially with the number of sequences *n*. Consequently, the computation time for computing an (n−1)-D MSA is negligible compared to that for an *n*-D MSA. Based on this observation, we designed the workflow of the proposed method (Fig. 4). In addition to the DP matrices in PSA, the h-score estimator leverages the results of the (n−1)-D MSA to compute h-scores for the *n*-D MSA. The *i*-D MSA task takes the first *i* sequences as input, where 1≤i≤n. Let $p^{n}$ denote a node *p* in the *n*-D task.

There are two primary advantages to using recursive MSA for h-score computation. (1) When the target node *p* is found in the (n−1)-D closed list $L_{\text{closed}}^{n-1}$, the method reduces PSA score accesses by directly using the g-score $g_{p}^{n-1}$ in the closed list. (2) The (n−1)-D closed list provides the precise score of the shortest path, whereas the PSA scores may underestimate the true path score. The best h-score estimator should return a score as close to the actual one as possible. Therefore, leveraging the (n−1)-D MSA results provides better heuristic estimates.

For a node $p^{n}$ in the *n*-D MSA task, its h-score $h_{p}^{n}$ is computed according to Equation (6):


(6) $$h_{p}^{n}=\{\begin{array}{cc} g_{p}^{n-1}+\sum_{i=1}^{n-1}c(A^{*}({\hat{s}}_{i},{\hat{s}}_{n})), & \text{if}\;p^{n-1}\in L_{\text{closed}}^{n-1},\\ \sum_{i=1}^{n-1}\sum_{j=i+1}^{n}c(A^{*}({\hat{s}}_{i},{\hat{s}}_{j})), & \text{otherwise}. \end{array}$$

If $p^{n-1}$ is expanded in the (n−1)-D MSA task, then $h_{p}^{n}$ can be computed with the (n−1)-D g-score read from the closed list. Otherwise, $h_{p}^{n}$ is computed by summing the all-to-all PSA scores.

**Figure 5:**
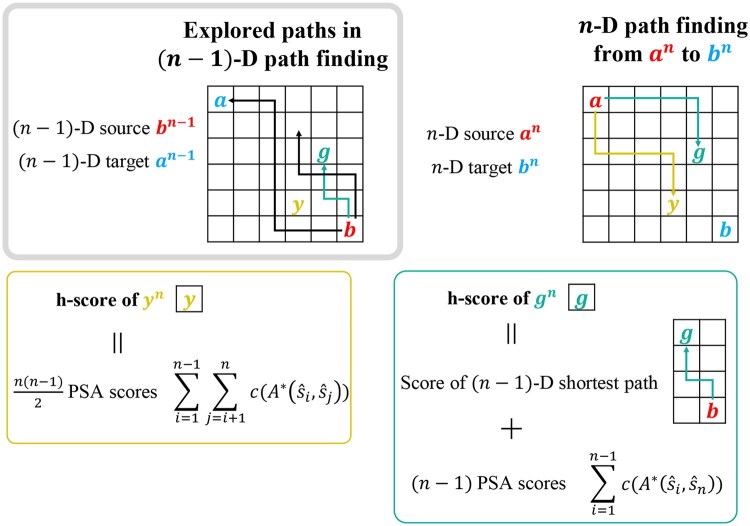
Calculating the h-score in recursive MSA. Because the green node is explored in the (n−1)-D path finding, we compute the h-score by summing the score of the (n−1)-D shortest path and the (n−1) PSA scores. The yellow node is not explored in the (n−1)-D path finding, thus the h-score is computed only with the all-to-all PSA scores.

Figure 5 shows how to calculate the h-score in the recursive MSA method. Because the paths to estimate scores always end at the target node, we search the (n−1)-D shortest path reversely, i.e. from the target node to the source node. When we estimate the h-score of a node in *n*-D path finding, we search its corresponding node in the (n−1)-D closed list. The h-score is calculated by summing the g-score of the found node and the (n−1) PSA scores, like the green node $g^{n}$ in Fig. 5. However, the search could fail because the closed list only contains the expanded nodes in the (n−1)-D path finding. In that case, the h-score is computed normally using the CL lower bound with all-to-all PSA scores, like the yellow node $y^{n}$ in Fig. 5.

### 3.2 Anytime column search tailored for MSA

We develop an anytime MSA method by combining A* search and a variant of ACS tailored for MSA tasks. ACS continuously outputs intermediate alignments of high accuracy, ensuring that useful results are available even if the program is terminated accidentally. Furthermore, the method initializes and dynamically updates a score upper bound using the intermediate MSA results. Incorporating score upper bounds improves performance by pruning unpromising nodes before they are added to the open list.

Algorithm 2 Proposed anytime A* algorithm for MSA

**Input:** A set of sequences $S=\{s_{1},s_{2},\ldots ,s_{n}\}$, ACS bin count α, ACS iterations β, and A* search iterations γ.

**Output:** The best possible alignment *A*[*n*][*l*].

1: Minimal alignment score $c_{A}=+\infty$

2: Create the source node $p_{s}$ with all-zero coordinates $0_{n}$

3: Open lists $L_{\text{open}}[\alpha ]=\{\{p_{s}\},\emptyset ,\ldots ,\emptyset \}$ // α priority queues

4: Closed list $L_{\text{closed}}=\emptyset$ // unordered map

5: **while**  $L_{\text{open}}\neq \emptyset$  **and**  $L_{\text{open}}.\text{top}().\text{fscore}<c_{A}$  **do** 

6:  **for** bin index j=0→(α−1)  **do** 

7:   **for** iteration i=0→(β−1)  **do** 

8:    Current node $p=L_{\text{open}}[j].\text{pop}()$

9:    ExpandNode(*p*)

10:   **end for** 

11:  **end for** 

12:  **for** iteration i=0→(γ−1)  **do** 

13:   $p=L_{\text{open}}.\text{pop}()$ // lowest f-score in all bins

14:   ExpandNode(*p*)

15: **end for** 

16: **end while** 

17: **return** *A*

18:

19: **procedure**  ExpandNode(*p*)

20:  **if**  p.is_target()  **and**  $p.\text{gscore}<c_{A}$  **then** 

21:   *A* = traceback(*p*)

22:   $c_{A}=p.\text{gscore}$

23:  **else if**  $p.\text{gscore}<L_{\text{closed}}.\text{find}(p).\text{gscore}$  **then** 

24:   $L_{\text{closed}}.\text{insert}(p)$

25:   **for** each *q* **in** neighbors of *p* **do** 

26:    **if**  $q.\text{gscore}<L_{\text{open}}.\text{find}(q).\text{gscore}$  **and**  $q.\text{gscore}<L_{\text{closed}}.\text{find}(q).\text{gscore}$  **and**  $q.\text{fscore}<c_{A}$  **then** 

27:     $L_{\text{open}}.\text{insert}(q)$

28:    **end if** 

29:   **end for** 

30:  **end if** 

31: **end procedure** 

Algorithm 2 presents the proposed anytime A* algorithm for MSA. The outer while loop consists of the ACS procedure (lines 6–11) and the A* search procedure (lines 12–15). There are two key modifications compared to the standard A* algorithm. (1) The open list data structure uses α bins instead of a single list to store the nodes to expand. (2) A variable $c_{A}$ tracks the minimal alignment score, which is used to verify the termination condition and prune unpromising nodes. We explain the details in the following two paragraphs.

The open list data structure uses α bins instead of a single list. The proposed method reaches the target node rapidly by distributing nodes into bins based on their g-scores. Assuming the lowest possible MSA score is $c_{l}$, the first bin stores the nodes with g-scores in the range $\left[0,c_{l}/\alpha \right)$. The second bin covers $\left[c_{l}/\alpha ,2c_{l}/\alpha \right)$, and the remaining bins are defined similarly. Especially, the last bin covers $\left[(\alpha -1)c_{l}/\alpha ,+\infty \right)$. The CL lower bound of the input sequences is used as $c_{l}$. By prioritizing deeper nodes, i.e. nodes with higher g-scores, in latter bins, the algorithm reaches the target node more quickly than the standard A* search. Our approach is extended from the existing A* + ACS method, which was designed for uniform cost search problems and assigns a bin to each level of the search tree. However, this strategy becomes infeasible for MSA due to the varying scores in the substitution matrices. Therefore, we redesign the rules to adapt to MSA: we precompute the lowest possible MSA score and assign a bin to the corresponding score range.

Unlike A* search, which terminates immediately upon reaching the target node, ACS may reach the target via a suboptimal path. We thus maintain a variable $c_{A}$ to store the minimal alignment score found so far. The proposed method realizes an anytime capability because it outputs a new alignment each time a better alignment is found and updates $c_{A}$ accordingly. When $c_{A}$ is lower than the lowest f-score among all remaining nodes in the open list, it indicates that the most recent alignment is optimal, and the search terminates. Practically, we initialize $c_{A}$ by obtaining the first MSA with a greedy path-finding method, which repeatedly selects the neighbor with the lowest f-score until reaching the target.

Algorithm 2 takes three additional parameters, α, β, and γ, along with the input sequences. These parameters control the number of iterations for ACS and A* search. We empirically fix them at α=4, β=10, and γ=100, which leads to good performance across most test cases.

### 3.3 Memory-bound strategy

Algorithm 3 Proposed memory-bound strategy

**Input:** Bin count α, open lists $L_{\text{open}}[\alpha ]$, closed list $L_{\text{closed}}$, memory threshold $t_{m}$, and minimal alignment score $c_{A}$.

**Output:** Updated open lists $L_{\text{open}}$ and closed list $L_{\text{closed}}$.

1: **while**  $L_{\text{open}}.\text{size}+L_{\text{closed}}.\text{size}>t_{m}$  **do** 

2:  k= index of the first non-empty bin

3:  p= node with the highest f-score in $L_{\text{open}}[k]$

4:  // reexp_fscore is used when re-expanding the node

5:  **if**  $p.\text{fscore}<L_{\text{closed}}[p.\text{parent}].\text{reexp}\_\text{fscore}$  **then** 

6:   $L_{\text{closed}}[p.\text{parent}].\text{reexp}\_\text{fscore}=p.\text{fscore}$

7:  **end if** 

8:  **if**  $L_{\text{open}}.\text{find}(p.\text{parent})$  **then** 

9:   **if**  $p.\text{fscore}<L_{\text{open}}.\text{get}\_\text{fscore}(p.\text{parent})$  **then** 

10:    $L_{\text{open}}.\text{set}\_\text{fscore}(p.\text{parent},p.\text{fscore})$

11:   **end if** 

12:  **else** 

13:   Create *q* with $L_{\text{closed}}[p.\text{parent}]$ // using reexp_fscore

14:   **if**  $q.\text{fscore}<c_{A}$  **then** 

15:    $L_{\text{open}}.\text{insert}(q)$

16:   **end if** 

17:  **end if** 

18: **end while** 

19: **return**  $L_{\text{open}}$ and $L_{\text{closed}}$

Exponentially growing memory consumption is the major obstacle to solving large-scale exact MSA problems. Because the open list constitutes the primary memory bottleneck, we develop a memory-bound strategy that removes unpromising nodes from the open list to reduce memory usage. To ensure the correctness of the final alignment, information of a removed node is stored in its parent, allowing the algorithm to retrieve pruned nodes when there is sufficient memory space.

Algorithm 3 illustrates the proposed memory-bound strategy. After inserting the neighbors of a node into the open list (Algorithm 2, lines 25–29), the algorithm verifies whether the combined size of the open and closed lists exceeds a predefined memory threshold $t_{m}$ (Algorithm 3, line 1). If the memory limit is exceeded, the method removes the least promising node in the first non-empty open list bin, i.e. the bin storing nodes with the smallest g-scores. Unlike other path-finding methods with explicit graph representations, the proposed method searches for a parent in the closed list because MSA problems are defined over implicit graphs. The reexp_fscore variable, representing the f-score for re-expanding, in a closed list node indicates the potential of its removed children and is used as the f-score when reinserting the node into the open list (Algorithm 3, line 5 and line 13).

The open list size remains unchanged when the algorithm removes a node and inserts its parent. Therefore, the algorithm loops until one of the following occurs: (1) The parent is already present in the open list. (2) The f-score of the parent is higher than $c_{A}$, the minimal alignment score found so far. The memory-bound strategy mitigates the issue of exponential memory growth, even with a relatively small memory threshold like 4 GB. However, it introduces a trade-off between memory usage and computational efficiency because frequent updates and reinsertions of parent nodes incur extra overhead.

In addition to managing the open list, the algorithm prunes unnecessary nodes in the closed list every time it finds a better alignment. Specifically, the algorithm traverses the closed list and computes the sum of the g-score and h-score for each node. The node is removed from the closed list if the sum is higher than $c_{A}$. This traversal takes negligible time compared to the overall search process, but it frees reusable memory space for the upcoming nodes, improving the algorithm’s efficiency.

### 3.4 Extension to affine gap penalties

To compute affine gap penalties during the search and to reconstruct the alignment after reaching the target, the data structure of an open list node is modified. Specifically, the traceback information is changed from storing the parent direction to storing the gap lengths. The other three elements, (1) coordinate array, (2) g-score, and (3) f-score, remain the same as in the linear cases. The gap lengths are essential not only for reconstructing the final alignment but also for calculating the affine gap penalty. They are represented as an integer array that tracks the length of the current gap for each sequence. Assuming $c_{i}$ is the coordinate of the *i*-th sequence, the gap length $m_{i}$ of the *i*-th sequence corresponds to the gap ends at $c_{i}$. For the example in Fig. 1, the coordinates and the gap lengths of the fourth column in the output alignment are {2,2,4,4} and {2,2,0,0}. Similarly, the gap lengths of the fifth and the seventh columns are {3,3,1,0} and {1,1,1,0}, respectively. Correspondingly, a closed list node also stores the g-scores for each combination of gap lengths for pruning and backtracking.

The expand_node() function in Algorithm 2 is also updated for affine gap penalties. A major difference is finding elements in the open and closed lists (lines 23 and 26). An element in the closed list is accessed in the format of $L_{\text{closed}}[c][m]$, where coordinates $c=\{c_{1},c_{2},\ldots ,c_{n}\}$ is the primary key and gap lengths $m=\{m_{1},m_{2},\ldots ,m_{n}\}$ is the secondary key. In practice, the closed list is implemented with a nested unordered map. Similarly, both the coordinates and gap lengths are used to find a node in the open list. Next, computing the g-score and h-score (line 26) is more complex than that in the linear cases because gap lengths are involved. Take the alignment in Fig. 1 as an example: For the fifth column, the coordinates and gap lengths are {2,2,4,5} and {3,3,1,0}, respectively. The penalty of sequences 1 and 4 is $p_{\text{aff}}^{1,4}=e$ because it is a gap extension in sequence 1. Similarly, $p_{\text{aff}}^{3,4}=o+e$ because it is a gap opening in sequence 3, and $p_{\text{aff}}^{1,3}=0$ because a pair of two gaps is ignored. Note that the method computes the increment of the gap penalty, i.e. $p_{\text{aff}}^{1,4}=e$ rather than $p_{\text{aff}}^{1,4}=o+3\times e$, because the g-score should be updated instantly for each neighboring node.

The number of possible combinations of gap lengths is larger than that of the parent directions. For an *n*-D node with coordinates c, the gap length in the *i*-th sequence ranges in $\left[0,c_{i}+1\right]$. Therefore, the number of possible combinations is $\prod_{i=1}^{n}\left(c_{i}+1\right)-1$. To reconstruct the alignment, each possible combination of gap lengths is considered a parent direction. The number of such combinations is $l^{n}$ for the target node. Thus, the workload of RAM-MSA is $O(l^{nl})$ for affine gap penalties, while it is $O(2^{nl})$ for linear gap penalties.

## 4 Results

We conducted the experiments with Ubuntu 22.04.5 LTS, an AMD Ryzen 5 36 006-core processor, and 32 GB RAM. The proposed RAM-MSA method was compared with four open-source baseline methods. Three widely used heuristic MSA methods are included: (1) MAFFT (Katoh and Standley 2013), (2) MSAProbs (Liu *et al.* 2010), and (3) MUSCLE (Edgar 2004). We also compared our method with (4) PA-star2, the state-of-the-art parallel exact MSA method (Sundfeld *et al.* 2018, 2025). Table 1 lists the capabilities of the evaluated methods. The experiments of PA-star2 were parallelized with 12 threads.

**Table 1:** Evaluated methods and their capabilities, including support for linear gap penalties, affine gap penalties, and the generation of algorithmically optimal alignments.

Feature	RAM-MSA	PA-star2	MAFFT	MSAProbs	MUSCLE
Linear	Yes	Yes	No	No	No
Affine	Yes	No	Yes	Yes	Yes
Optimal	Yes	Yes	No	No	No

We evaluated the methods on three benchmarks. The first is a customized benchmark adopted from the PA-star2 study, consisting of 82 sequence sets without reference alignments. The other two benchmarks provide structurally-derived reference alignments: BAliBASE RV11 (Thompson *et al.* 1999), which includes 29 sequence sets, and the SABRE Twilight Zone benchmark (Van Walle *et al.* 2005), which includes 54 sequence sets. The sequence sets are divided into groups in our evaluation, as shown in Table 2. The benchmarks are divided according to what the exact methods can handle. For example, under linear gap penalties, RAM-MSA successfully computed exact alignments on BAliBASE RV11 Group 1 to 3 and produced anytime results for Group 4, whereas PA-star2 failed on Group 3 & 4.

**Table 2:** Benchmarks and their respective groups of sequence sets.

Benchmark	Sequence sets	RAM-MSA	PA-star2	MAFFT	MSAProbs	MUSCLE
Customized (Linear)	Group 1: 81 sets other than 1pamA (n∈[3,6],l∈[58,994])	✓	✓	✗	✗	✗
	Group 2: 1pamA (n=5,l=572)	✓	✗	✗	✗	✗
BAliBASE RV11 (Linear/Affine)	Group 1: BBS110{01, 09, 21, 22, 25, 29} (n=4,l∈[69,126])	✓/✓	✓/✗	✗/Δ	✗/Δ	✗/Δ
	Group 2: BBS110{03, 04, 08, 10, 12, 13, 15, 17, 24} (n∈[4,5],l∈[65,523])	✓/Δ	✓/✗	✗/Δ	✗/Δ	✗/Δ
	Group 3: BBS110{06, 11, 14} (n∈[5,8],l∈[233,618])	✓/Δ	✗/✗	✗/Δ	✗/Δ	✗/Δ
	Group 4: BBS110{02, 05, 07, 16, 18, 19, 20, 23, 26, 27, 28} (n∈[7,14],l∈[85,2850])	Δ/Δ	✗/✗	✗/Δ	✗/Δ	✗/Δ
SABRE Twi (Affine)	Group 1: twi_{009, 011, 022, 027, 028, 029, 034, 037, 043, 050, 054, 057, 059, 075, 088, 099, 102, 103, 110} (n∈[3,4],l∈[44,261])	✓	✗	Δ	Δ	Δ
	Group 2: twi_{026, 030, 038, 041, 049, 051, 053, 056, 060, 061, 063, 065, 066, 067, 068, 069, 070, 083, 086, 087, 089, 091, 094, 095, 096, 098, 100, 107, 109, 111, 112, 113, 114, 115, 116} (n∈[3,19],l∈[152,2675])	Δ	✗	Δ	Δ	Δ

The benchmarks are divided according to what the exact methods can handle: ✓ indicates exact alignments, Δ represents anytime or approximate alignments, and ✗ denotes failure. For example, on BAliBASE RV11 Group 2, RAM-MSA (✓/Δ) computed an exact alignment under linear gap penalties and produced anytime alignments under affine gap penalties.

The objective function of RAM-MSA and PA-star2 was the SP score. For problems under linear gap penalties, both exact methods used the PAM250 substitution matrix. To align with the settings of PA-star2, the matrix scores were adjusted to non-negative by adding an offset of 17. To improve performance from a biological perspective, we have tuned gap penalty parameters based on the reference alignments. The linear gap penalty was set to 12, where lower alignment scores indicate better alignment quality. RAM-MSA additionally supports affine gap penalties. We evaluated RAM-MSA with BLOSUM62 under affine gap penalties, with the gap open and extension penalties set to 9.5 and 2.0, respectively. In affine cases, higher alignment scores indicate better alignment quality.

Three metrics were used to evaluate the MSA methods: objective score, SP accuracy, and total column (TC) accuracy. The objective score evaluates algorithmic optimality and is computed from the alignment score (i.e. the SP score) using the substitution matrix and gap lengths. SP accuracy and TC accuracy, in contrast, are computed with the reference alignments. SP accuracy is defined as the ratio of correctly aligned symbol pairs between the output alignment and the reference alignments to the total number of aligned symbol pairs in the reference alignments. TC accuracy is defined as the ratio of correctly aligned columns between the output alignment and the reference alignments to the total number of columns in the reference alignments.

The metrics are defined in Equations (7) to (9).


(7) $$\text{Obj}.\;\text{score}=\{\begin{array}{c} \frac{\text{test}\;\text{score}}{\;\text{exact}\;\text{score}},\text{if}\;\text{higher}\;\text{is}\;\text{better}\;(\text{affine})\\ \frac{\text{exact}\;\text{score}}{\text{test}\;\text{score}},\text{if}\;\text{lower}\;\text{is}\;\text{better}\;(\text{linear}) \end{array}$$

(8) $$\text{SP}\;\text{accuracy}=\frac{\#\text{correctly}\;\text{aligned}\;\text{symbol}\;\text{pairs}}{\#\text{symbol}\;\text{pairs}\;\text{in}\;\text{reference}\;\text{alignments}}.$$

(9) $$\text{TC}\;\text{accuracy}=\frac{\#\text{correctly}\;\text{aligned}\;\text{columns}}{\#\text{columns}\;\text{in}\;\text{reference}\;\text{alignments}}.$$

### 4.1 Algorithmic optimality

We evaluated the algorithmic optimality of RAM-MSA and the heuristic methods using the objective score of their output alignments. The average objective score is reported for each benchmark. Table 3 presents the results under affine gap penalties. We omitted the comparison under linear gap penalties because only exact methods are valid, and both generate exact results. Note that we only report the results for alignments successfully computed by RAM-MSA, which were BAliBASE RV11 Group 1 and SABRE Twilight Zone Group 1. Evaluating algorithmic optimality is infeasible when exact methods run out of memory because no reference alignments exists for the maximum possible objective score. We configured MSAProbs to use the same substitution matrix and gap penalties as RAM-MSA. MAFFT and MUSCLE used their default settings because their parameters were hard-coded in their programs. Furthermore, they had additional parameters beyond gap penalties that adversely affected the objective scores.

**Table 3:** Algorithmic optimality of RAM-MSA under affine gap penalties.

Benchmark	Method	Time (s)	Objective score
BAliBASE RV11	RAM-MSA	3367.21	1.00 (@161.84)
(Group 1)	PA-star2	N/A	N/A
	MAFFT	0.64	0.49 (−159.14)
	MSAProbs	0.17	0.73 (33.65)
	MUSCLE	0.18	0.82 (65.83)
SABRE Twi	RAM-MSA	9934.74	1.00 (−129.00)
(Group 1)	PA-star2	N/A	N/A
	MAFFT	2.37	0.02 (−584.76)
	MSAProbs	0.29	0.65 (−285.37)
	MUSCLE	0.38	0.63 (−299.16)

Higher scores indicate better accuracies. The values in the parentheses denote the objective scores before normalization. Note that PA-star2 did not support affine gap penalties.

Since some objective scores were negative prior to normalization, we applied an offset using the score of the unaligned input sequences. The objective scores were subsequently normalized to the range [0, 1], where 0 corresponds to the score of the unaligned input sequences.

On the BAliBASE RV11 and SABRE Twilight Zone benchmarks, RAM-MSA generated exact alignments for 6 and 18 instances, respectively. The computation time was relatively longer than that of the heuristic methods. MSAProbs computed the results rapidly compared to the exact MSA methods by sacrificing the algorithmic accuracy. The highest objective scores among the heuristic methods were 0.82 and 0.65 on BAliBASE RV11 and SABRE Twilight Zone, respectively. MAFFT achieved a noticeably lower objective score on SABRE Twilight Zone, likely because its default settings performed poorly on such highly divergent sequences.

### 4.2 Exact search efficiency

We compared the exact search efficiency of RAM-MSA with the state-of-the-art PA-star2 method. Table 4 shows the time and memory usage of exact search progresses of RAM-MSA and PA-star2 under linear gap penalties on the customized benchmark and the BAliBASE RV11 benchmark. RAM-MSA completed two groups (82 sequence sets) in the customized benchmark and three groups (18 sequence sets) in the BAliBASE RV11 benchmark, whereas PA-star2 completed one group (81 sets) and two groups (15 sets).

**Table 4:** Exact search efficiency of RAM-MSA under linear gap penalties.

Sequence set	Method	Time (s)	Peak RAM (GB)
Customized	RAM-MSA	13 364.03	13.12
(Group 1)	PA-star2	1546.55	20.99
Customized	RAM-MSA	29 377.69	25.97
(Group 2)	PA-star2	N/A	N/A
BAliBASE RV11	RAM-MSA	5497.88	8.67
(Group 1 & 2)	PA-star2	501.79	12.93
BAliBASE RV11	RAM-MSA	66 390.52	30.63
(Group 3)	PA-star2	N/A	N/A

N/A indicates a failure due to memory exhaustion.

On both benchmarks reported, RAM-MSA consumed less memory than PA-star2. Considering only the instances that PA-star2 completed, the memory usage of RAM-MSA was 62.51% and 67.05% compared to that of PA-star2 on the two benchmarks. A possible explanation is that RAM-MSA reduced its search space by leveraging intermediate alignment results, resulting in fewer node insertions into the open and closed lists. PA-star2 achieved a speedup of 8.6× and 10.96× over RAM-MSA on the customized benchmark and BAliBASE RV11, respectively. Nevertheless, the speedup was smaller than 12× despite using 12 threads for parallelization. This implies that a parallelized implementation of RAM-MSA could achieve a lower computation time than PA-star2.

RAM-MSA completed the search process within 32 GB RAM using the memory-bound strategy on the instances that PA-star2 failed. The instance in the customized benchmark had (n,l)=(5,572). The three instances in the BAliBASE RV11 had (n,l)=(8,541),(5,233),and (6,618). To determine the scalability limit of RAM-MSA on the test machine, we identified the minimum value of $w_{\text{linear}}={\;\text{log}\;}_{10}(2^{nl})$ among the instances that RAM-MSA failed to complete. Of note, the theoretical workload under linear gap penalties is $O(2^{nl})$. The minimum value of $w_{\text{linear}}={\;\text{log}\;}_{10}(2^{nl})$ among the four instances was 350.70. Nevertheless, RAM-MSA failed to generate the optimal alignment on an instance with $w_{\text{linear}}=204.70$, indicating that actual memory consumption varies by instance and depends heavily on the pruning strategy. The values of *l* and *n* correspond to the path length and the dimensionality of the search tree, respectively. The results suggested that the scalability under linear gap penalties is more sensitive to the dimensionality of the search tree.

Regarding the results in Table 3 under affine gap penalties, the theoretical workload of RAM-MSA is $O(l^{nl})$, whereas the actual memory consumption depends on the pruning strategy and is difficult to quantify precisely. The lowest observed $w_{\text{affine}}$ among uncompleted instances was 611.74, indicating that the program exhausts memory when $l^{nl}>10^{611.74}$. The results suggested that the scalability limit under affine gap penalties was more sensitive to the path length, i.e. the alignment length. RAM-MSA successfully computed more exact alignments in the linear cases than in the affine ones. As explained in Section 3.4, computing alignments under linear gap penalties is less complex and has a lower workload.

### 4.3 Anytime performance

**Figure 6:**
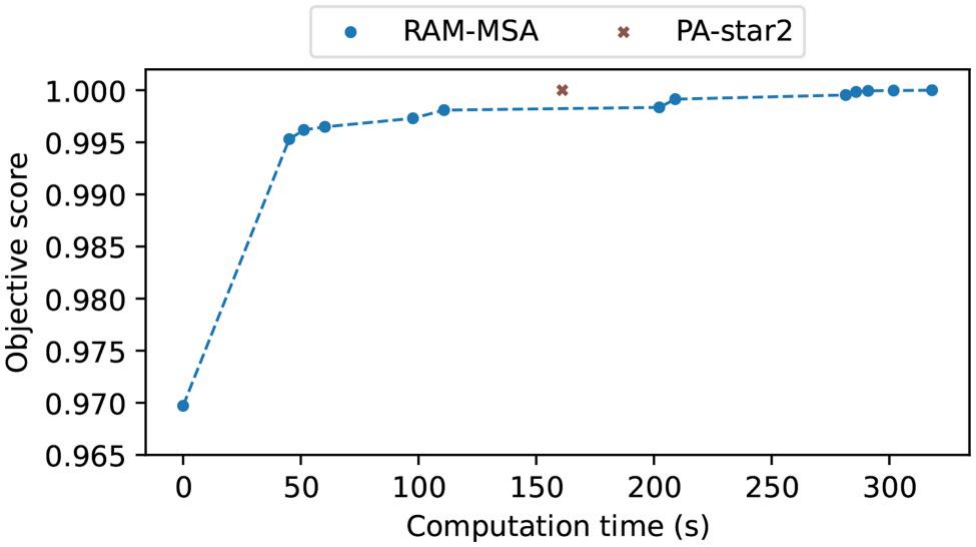
Anytime performance on the BAliBASE RV11 BBS11024 (n=4,l=385) under linear gap penalties. Higher objective scores indicate better alignment qualities. PA-star2 was parallelized with 12 threads, whereas RAM-MSA was a sequential method using one thread.

We demonstrate the anytime performance of the RAM-MSA method by illustrating how much time it has taken to generate the intermediate MSA results. Figure 6 shows the results on the BAliBASE RV11 BBS11024 sequence set under linear gap penalties. The first anytime result was produced by the greedy search in 0.006 seconds with an objective score close to 0.97. The second result was generated by the anytime feature using the extension of ACS in 46 seconds, with an objective score exceeding 0.995. PA-star2 generated the exact alignment in 162 seconds, whereas RAM-MSA generated the exact alignment in 319 seconds. The anytime feature performed well under linear gap penalties because it generated highly accurate results considerably faster than PA-star2.

**Figure 7:**
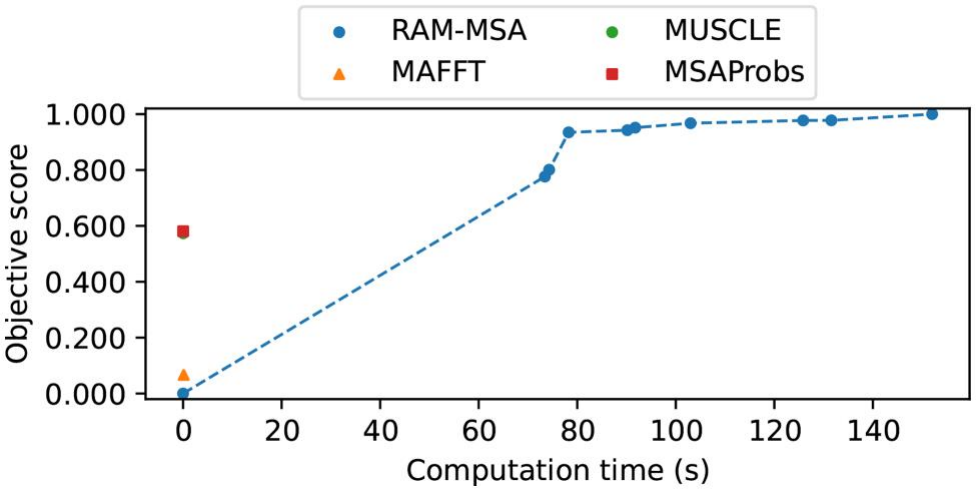
Anytime performance on the SABRE Twilight Zone twi_102 (n=3,l=197) under affine gap penalties. Higher objective scores indicate better alignment qualities from an algorithmic perspective. Note that PA-star2 did not support affine gap penalties.

Figure 7 demonstrates the results on the SABRE Twilight Zone twi_102 sequence set under affine gap penalties. The greedy search generated an alignment with an extremely low objective score, indicating that it performed worse under affine gap penalties. The second output achieved an objective score close to 0.8, which was better than the approximate results rapidly generated by the heuristic methods.

Note that RAM-MSA required extra time to expand all the promising nodes to guarantee the result’s optimality after finding the last MSA. It took 1841.75 seconds and 195.52 seconds to complete the search on BAliBASE RV11 BBS11024 and on SABRE Twilight Zone twi_102, respectively.

### 4.4 Ablation study

We conducted an ablation study on the four variants of the RAM-MSA method. The first variant was the standard A* method, against which we showed the speedups of the other variants. The remaining variants corresponded to our first three contributions. The second variant used the recursive MSA approach to enhance the h-score estimator by exploiting lower-dimensional MSA results. The third variant incorporated the ACS paradigm to continuously generate intermediate MSA results and dynamically update the score upper bound for node pruning. The fourth variant, the RAM-MSA method, integrated the memory-bound strategy to compute large-scale sequence sets with a limited memory space.

**Figure 8:**
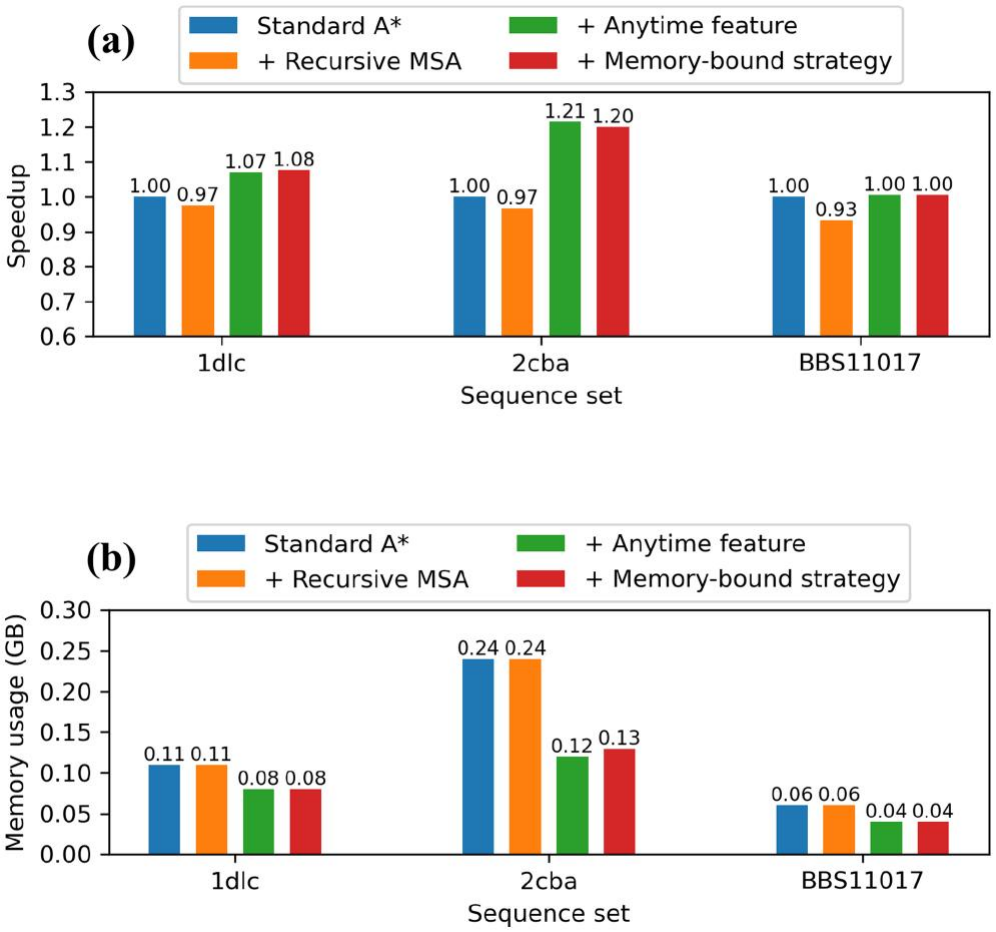
Ablation study of the RAM-MSA method regarding (a) speedup of computation time and (b) memory usage for customized 1dlc (n=4,l=590), customized 2cba (n=5,l=259), and BAliBASE RV11 BBS11017 (n=4,l=273) under linear gap penalties.

Figure 8 shows the results of the ablation study of RAM-MSA on customized 1dlc, 2cba, and BAliBASE RV11 BBS11017 sequence sets under linear gap penalties. In Fig. 8a, the recursive MSA method showed an insignificant impact on speedup and even slowed down the computation for some sequence sets. This phenomenon was possibly related to the usage of recursive MSA results. When the method found the recursive MSA result for a node *p*, it computed a better estimate for the path score, which helped prune more candidate nodes. However, most of the nodes without a corresponding recursive MSA result could have a worse estimate than node *n*, which gave *n* a low priority in the open list. When only a subset of the nodes achieved a better estimate, the speedup was restricted.

The third and fourth variants adopted the ACS paradigm to dynamically update the score upper bound. They achieved higher speedups than the second variant by pruning more nodes from the open list. The fourth variant periodically checked its memory usage, which had little effect on computation time.

The memory usage of the four variants is shown in Fig. 8b. In general, a variant with lower computation time also required less memory because fewer nodes were inserted into the open list and expanded. RAM-MSA used about half the memory required by the standard A* method on customized 2cba.

**Figure 9:**
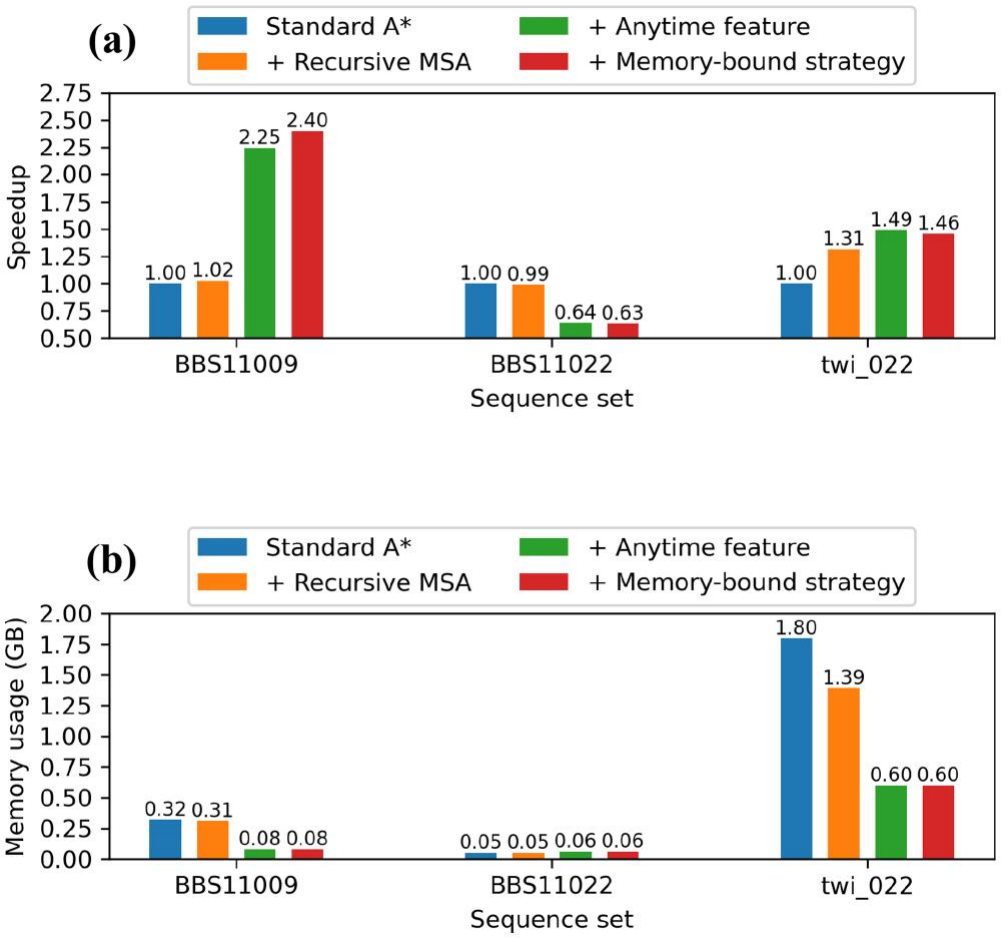
Ablation study of the RAM-MSA method regarding (a) speedup of computation time and (b) memory usage for BAliBASE RV11 BBS11009 (n=4,l=105), BAliBASE RV11 BBS11022 (n=4,l=69), and SABRE Twilight Zone twi_022 (n=4,l=100) under affine gap penalties.

The results of the ablation study under affine gap penalties are demonstrated in Fig. 9, including performance on BAliBASE RV11 BBS11009, BBS11022, and SABRE Twilight Zone twi_022. The recursive MSA method accelerated computation by a factor of 1.31 on twi_022. The anytime feature derived from the ACS paradigm achieved a speedup of 2.25× on BBS11009. However, computation slowed down on BBS11022 when the anytime feature was used. A potential reason is that very few nodes were pruned because no intermediate alignment other than the greedy result was generated during the search process. When comparing the third variant with standard A*, memory usage was reduced by approximately 75% and 66% on BBS11009 and twi_022, respectively.

### 4.5 Biological significance

The biological significance of the alignments generated by RAM-MSA and the baseline methods was evaluated using the reference alignments. We excluded the customized benchmark because no reference alignments are available. The average SP and TC accuracies of the exact results generated by RAM-MSA under affine and linear gap penalties are shown in Tables 5 and 6, respectively.

**Table 5:** Biological significance of RAM-MSA under affine gap penalties.

Benchmark	Method	Time (s)	SP	TC
BAliBASE RV11	RAM-MSA (affine)	3367.21	0.73	0.56
(Group 1)	PA-star2	N/A	N/A	N/A
	MAFFT	0.64	0.60	0.36
	MSAProbs	0.17	0.78	0.59
	MUSCLE	0.18	0.78	0.59
SABRE Twi	RAM-MSA (affine)	9934.74	0.31	0.18
(Group 1)	PA-star2	N/A	N/A	N/A
	MAFFT	2.37	0.16	0.06
	MSAProbs	0.29	0.32	0.19
	MUSCLE	0.38	0.31	0.19

The three heuristic methods used their default settings. Higher SP and TC values indicate better accuracies. Note that PA-star2 did not support affine gap penalties.

**Table 6:** Biological significance of RAM-MSA under linear gap penalties.

Benchmark	Method	Time (s)	SP	TC
BAliBASE RV11	RAM-MSA (linear)	71 888.40	0.46	0.24
(Group 1 & 2 & 3)	PA-star2	N/A	N/A	N/A
	MAFFT	1.97	0.40	0.22
	MSAProbs	1.03	0.71	0.50
	MUSCLE	1.07	0.72	0.52

The three heuristic methods used their default settings for affine gap penalties. Higher SP and TC values indicate better accuracies. The results of PA-star2 are invalid because it failed on BAliBASE RV11 Group 3.

In Table 5, RAM-MSA achieved higher SP and TC accuracies than MAFFT on both benchmarks. However, MSAProbs and MUSCLE performed better on BAliBASE RV11. On SABRE Twilight Zone, RAM-MSA achieved SP and TC accuracies of 0.31 and 0.18, respectively, which are comparable to the best results among the heuristic methods. The drawbacks of RAM-MSA are its non-negligible computation time and scalability limitations, arising from its focus on algorithmic optimality.

Of note, the heuristic methods used their default gap penalty settings, whereas RAM-MSA set the gap open and extension penalties to 9.5 and 2.0. We attempted to apply the gap penalties used by MAFFT and MSAProbs to RAM-MSA, but this resulted in worse accuracy than achieved with the current settings. This observation suggests that the optimal gap penalty settings likely differ between the exact and heuristic MSA methods. This is because the heuristic methods actually compute MSAs with several rounds of pairwise alignments. Furthermore, other parameters are involved in the computation, such as an offset value in MAFFT, meaning that accuracy does not depend solely on the gap open and extension penalties.

Although the exact methods computed algorithmically optimal alignments, the SP and TC accuracies were often lower than those of the state-of-the-art heuristic MSA methods. A potential reason is that the heuristic methods analyzed the input from a phylogenetic perspective to improve accuracy, such as computing a guide tree for the sequences.

Table 6 shows the biological significance of RAM-MSA under linear gap penalties. Of note, the three heuristic methods computed the alignments with their default settings for affine gap penalties. On BAliBASE RV11, RAM-MSA successfully computed more sequence sets in the linear cases than in the affine cases, but the SP and TC accuracies decreased gradually. The TC accuracy of RAM-MSA was 0.24, whereas MUSCLE achieved the highest TC accuracy of 0.52 among the baselines. Comparing the results of RAM-MSA in Tables 5 and 6, the affine gap penalties were more feasible than the linear ones for computing biologically significant MSA.

Table 7 shows the anytime performance of RAM-MSA on BAliBASE RV11 Group 2 to 4 and SABRE Twilight Zone Group 2 under affine gap penalties. RAM-MSA failed to compute the optimal MSA and instead generated only the anytime results for these groups. It was difficult to evaluate the algorithmic optimality of the anytime results because no exact optimal alignments exist. The SP and TC accuracies of RAM-MSA were relatively lower than those of the heuristic methods. The major reason is that the anytime performance declines when n>5. Specifically, RAM-MSA produced no intermediate output other than the greedy search result when n>5. Furthermore, the greedy search produced results of low accuracy.

**Table 7:** Anytime performance of RAM-MSA.

Benchmark	Method	Time (s)	SP	TC
BAliBASE RV11	RAM-MSA (affine)	5775.14	0.25	0.10
(Group 2 & 3 & 4)	PA-star2	N/A	N/A	N/A
	MAFFT	2.67	0.31	0.12
	MSAProbs	2.88	0.66	0.38
	MUSCLE	2.90	0.67	0.40
SABRE Twi	RAM-MSA (affine)	5534.33	0.08	0.08
(Group 2)	PA-star2	N/A	N/A	N/A
	MAFFT	4.82	0.05	0.03
	MSAProbs	2.00	0.23	0.11
	MUSCLE	3.17	0.23	0.10

The results show biological significance achieved under affine gap penalties. The three heuristic methods used their default settings. Higher SP and TC values indicate better accuracies. Note that PA-star2 did not support affine gap penalties.

**Figure 10:**
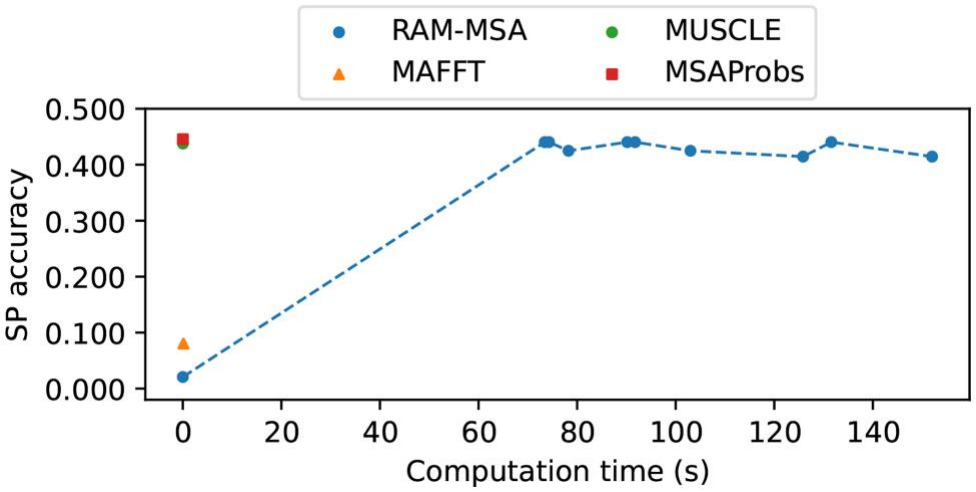
Anytime performance on the SABRE Twilight Zone twi_102 (n=3,l=197) under affine gap penalties. Higher SP accuracies indicate better alignment qualities from a biological perspective. Note that PA-star2 did not support affine gap penalties.

The anytime performance on the SABRE Twilight Zone twi_102 is shown in Fig. 10. Although the objective score increased to 1.00 as shown in Fig. 7, the SP accuracy showed no significant improvement. The second anytime output achieved an SP accuracy of 0.44, which was higher than the algorithmically optimal alignment outputted last with an SP accuracy of 0.41. The fluctuating results in Fig. 10 indicate that the algorithmic optimality diverges from biological correctness.

## 5 Discussion

We introduced RAM-MSA, an anytime memory-bounded method for exact MSA tasks. We proposed a recursive MSA approach to improve h-score calculation, combined A* search and a variant of ACS to enable the anytime feature, and developed a memory-bound strategy to compute large-scale MSA problems within a limited memory capacity. RAM-MSA supports affine gap penalties, whereas existing path-finding-based exact methods only support linear gap penalties. By comparing the exact alignments with structurally-derived reference alignments, this study provides insights into the limitations of exact MSA methods and highlights the need for improved objective functions that better capture biological relevance.

### 5.1 Key outcomes of RAM-MSA

Regarding algorithmic optimality, RAM-MSA generated optimal MSAs achieving an objective score of 1.00, whereas the highest objective score among the heuristic MSA methods was 0.82 on BAliBASE RV11 Group 1.

RAM-MSA leverages (n−1)-D MSA results to guide *n*-D tasks and incorporates the ACS paradigm to realize the anytime property while accelerating computation. RAM-MSA achieved a speedup of up to 2.25× and reduced memory usage to 25% with these two features on BAliBASE RV11 BBS11009.

In addition to the memory usage reduction induced by the anytime feature, the memory-bound strategy further enabled RAM-MSA to complete an exact MSA within a limited memory space. Specifically, RAM-MSA computed the exact MSA on BAliBASE RV11 BBS11006 with (n,l)=(8,541), on which the existing exact MSA method failed.

The anytime feature also enabled RAM-MSA to rapidly generate an initial output with relatively high accuracy under linear gap penalties. On BAliBASE RV11 BBS11024, RAM-MSA achieved an objective score of over 0.96 within 1 second, whereas the optimal MSA required over 300 seconds to generate.

### 5.2 Limitations of RAM-MSA

RAM-MSA addresses exact MSA problems primarily from an algorithmic perspective. As a result, the output alignments may differ substantially from the reference alignments because optimizing the objective function does not necessarily guarantee biological correctness. For instance, RAM-MSA achieved an SP accuracy of 0.73 on BAliBASE RV11 Group 1, while MSAProbs achieved an SP accuracy of 0.78 with its heuristic output. To enhance the accuracy of exact methods, an objective function that more faithfully captures the biological relationships among sequences is required.

The scalability of RAM-MSA remains limited despite its memory-bounded extension because memory consumption grows exponentially with both *n* and *l*. The program ran out of memory on a machine with 32 GB of RAM when processing a task with $l^{nl}>10^{611.74}$ under affine gap penalties, and when $2^{nl}>10^{204.70}\approx 2^{680.00}$ under linear gap penalties. The scalability limit is higher under affine gap penalties than under linear gap penalties because the pruning strategy omits a large portion of nodes in the affine case. In practice, actual memory usage also depends on the pruning strategy, beyond what the theoretical workload predicts.

### 5.3 Future work

In future work, we aim to leverage multi-core CPUs by parallelizing the proposed method to improve computational efficiency. To accelerate node expansion when handling a large number of sequences, we further plan to explore GPU platforms (Okada *et al.* 2015, Sundfeld *et al.* 2025). Regarding the anytime parameters, we will perform systematic parameter tuning rather than directly adopting the settings from previous studies. Additionally, the greedy search under affine gap penalties requires improvement.

## Data Availability

The data and code underlying this article are available in RAM-MSA, at https://github.com/luxwj/RAM-MSA.
